# Brain-muscle interplay during endurance self-paced exercise in normobaric and hypobaric hypoxia

**DOI:** 10.3389/fphys.2022.893872

**Published:** 2022-08-25

**Authors:** Thomas Rupp, Jonas J. Saugy, Nicolas Bourdillon, Grégoire P. Millet

**Affiliations:** ^1^ LIBM, Inter-university Laboratory of Human Movement Science, University Savoie Mont Blanc, Chambéry, France; ^2^ ISSUL, Faculty of Biology and Medicine, Institute of Sport Sciences, University of Lausanne, Lausanne, Switzerland

**Keywords:** altitude, cerebral oxygen delivery, near-infrared spectroscopy, pacing strategies, performance, time-trial exercise

## Abstract

**Purpose:** Hypoxia is one major environmental factor, supposed to mediate central motor command as well as afferent feedbacks at rest and during exercise. By using a comparison of normobaric (NH) and hypobaric (HH) hypoxia with the same ambient pressure in oxygen, we examined the potential differences on the cerebrovascular and muscular regulation interplay during a self-paced aerobic exercise.

**Methods:** Sixteen healthy subjects performed three cycling time-trials (250 kJ) in three conditions: HH, NH and normobaric normoxia (NN) after 24 h of exposure. Cerebral and muscular oxygenation were assessed by near-infrared spectroscopy, cerebral blood flow by Doppler ultrasound system. Gas exchanges, peripheral oxygen saturation, power output and associated pacing strategies were also continuously assessed.

**Results:** The cerebral oxygen delivery was lower in hypoxia than in NN but decreased similarly in both hypoxic conditions. Overall performance and pacing were significantly more down-regulated in HH *versus* NH, in conjunction with more impaired systemic (e.g*.* saturation and cerebral blood flow) and prefrontal cortex oxygenation during exercise.

**Conclusions:** The difference in pacing was likely the consequence of a complex interplay between systemic alterations and cerebral oxygenation observed in HH compared to NH, aiming to maintain an equivalent cerebral oxygen delivery despite higher adaptive cost (lower absolute power output for the same relative exercise intensity) in HH compared to NH.

## 1 Introduction

Hypoxia is one major environmental factor of interest when considering training and performance at altitude since it mediates cerebral oxygen supply and central motor command as well as afferent feedbacks at rest and during exercise (Amann et al., 2007; Subudhi et al., 2009; Subudhi, Panerai and Roach, 2010; Vogiatzis et al., 2011; Verges et al., 2012; Goodall, Twomey and Amann, 2014; Marillier et al., 2021). Only a few studies investigated self-paced exercise when arterial oxygenation is manipulated, suggesting marginal differences (Clark et al., 2007; Beidleman et al., 2014) or similar trends (Amann et al., 2006; Périard and Racinais, 2016) in pacing between normoxia and hypoxia, despite reduced average power output in the latter. A major concern regarding these studies is that subjects were exposed to simulated altitude for only 5–40 min before time-trials. Oxygen delivery to the tissues (e.g. muscle, brain) progressively evolves in the first hours of exposure to hypoxia (e.g. time delay in changes between arterial and cerebral oxygenation (Rupp and Leti, 2013), and from a practical point of view, exposure time preceding training/competing at altitude is usually longer and prone to influence how the practitioner may feel (Luks, 2015). Altogether, pacing strategies adopted after a more prolonged exposure period (e.g. 24-h) are relevant and may strongly differ from what has been observed with short-term acute exposure (Tucker, 2009). Discrepancies in the literature regarding altered pacing in hypoxia may also result from the type of hypoxia (normobaric hypoxia NH *versus* hypobaric hypoxia HH) used in these models. Indeed, numerous disparities have been recently found in subjects resting and exercising at a given pressure of inspired oxygen (P_i_O_2_), but among NH or HH conditions (Faiss et al., 2013). For instance, a combination of a higher tidal volume and lower respiratory frequency leading to a higher minute ventilation in NH compared to HH (Conkin and Wessel, 2008) has been reported and markers of oxidative stress have recently been shown lower in NH compared to HH (Faiss et al., 2013; Ribon et al., 2016). The mechanisms underlying these slight physiological differences are not so clear yet. Nevertheless, we have just shown that such differences are associated with impaired global performance in HH compared to NH on a 250-kJ cycling time-trial at 3,450 m (Saugy et al., 2016). However, the extent to which underlying physiological responses driving pacing strategies would be differentially affected in HH *versus* NH remains unknown.

We therefore examined if there are differences in cerebrovascular regulation and muscular activation in relation to oxygen consumption, arterial saturation, cerebral and muscle hemodynamics and deoxygenation, subjective discomfort, RPE and how it would influence or be influenced by different pacing strategies during a 250-kJ cycling time-trial conducted after 24 h: 1) in normobaric hypoxia (NH) at a simulated altitude of 3,450 m, 2) in hypobaric hypoxia (HH) at a terrestrial altitude of 3,450 m and, 3) in normobaric normoxia (NN) as a control condition. We hypothesized that there might be subtle differences in the regulation of oxygen delivery to the brain between NH and HH, inducing different pacing strategies and consequently, the supposed better endurance performance in NH, when compared to HH. It was also anticipated that SpO_2_ and cerebral hemodynamics would be more affected in HH, compared to NH, leading to suboptimal pace management and a greater performance decrement. By comparing cerebrovascular and brain *versus* muscle deoxygenation time-course during self-paced endurance exercise in NH *versus* HH, we aim to better understand how exercise is regulated in hypoxia and to explore new mechanisms on the interplay between cerebrovascular and muscular regulation in humans.

## 2 Methods

Experimental design and partial parts of the methods have already been presented in a previous paper (Saugy et al., 2016) focusing on global exercise performance in HH *versus* NH but with no mention to the present interests (*i.e.*, exercise intensity regulation and underlying cerebrovascular and muscular regulation). For the convenience of the reader, key methodological information is redefined in the present paper.

### 2.1 Subjects

Sixteen healthy, trained male subjects volunteered to participate to this study (mean ± SD; age 34.7 ± 9.5 years, body weight 75.2 ± 7.2 kg, height 180 ± 6 cm, maximal oxygen consumption VO_2max_ 60.2 ± 9.9 ml kg^−1^ min^−1^). Participants were all experienced (recreational or competitive but not elite) cyclists. Written informed consent was obtained from each participant before participation. Subjects were non-smokers, and neither acclimatized nor recently exposed to altitude. All procedures conformed to the standards set by the *Declaration of Helsinki* and the study was approved by a Medical Ethics Committee (Commission Cantonale Valaisanne d’Ethique Médicale, CCVEM; Agreement 051/09).

### 2.2 Experimental design

The experimental design consisted in a preliminary visit and three testing sessions. During the first meeting to the laboratory, subjects completed the baseline anthropological measurements and filled the consent form. Participants then performed 1) a maximal incremental exercise test (F_i_O_2_: 0.21; 60 W + 30 W min^−1^) to determine VO_2max_ and peak workload on a braked cycle ergometer including a Powertap sensor (Cycleops IC 400 Pro, Madison, Wisconsin, United States); and 2) a familiarization test with the 250-kJ time-trial on the same ergocycle.

The experimental design was then composed of three different sessions in a randomized order separated by at least 12 days. One session was performed in a hypobaric hypoxia (HH) environment at the Altitude Research Station in Jungfraujoch (3,450 m, F_i_O_2_ of 20.9%, BP of 481.8 ± 4.7 mmHg, P_i_O_2_ of 90.9 ± 1.0 mmHg, temperature of 21.3 ± 0.6°C, humidity of 45.1 ± 8.3%). Two sessions were conducted in a hypoxic chamber (ATS Altitude, Sydney, Australia) built in our laboratory (Sion, 485 m, Switzerland). Temperature inside the chamber was maintained constant by an internal air conditioning system. One of the two sessions completed in the chamber was performed in normobaric hypoxia (NH) with a F_i_O_2_ of 13.6% (BP of 715.8 ± 3.8 mm Hg, P_i_O_2_ of 91.0 ± 0.6 mmHg, a temperature of 22.7 ± 0.8°C, and a humidity of 41.0 ± 4.8%) corresponding to a simulated altitude of 3,450 m. The other session was performed in normobaric normoxia (NN) with a F_i_O_2_ of 20.9% (BP: 718.1 ± 3.3 mmHg, P_i_O_2_ of 140.5 ± 0.6 mmHg, a temperature of 23 ± 1°C, and a humidity of 42.8 ± 4.4%.). These parameters were controlled regularly with an electronic device (GOX 100 oximeter, Greisinger, Regenstauf, Germany). In order to blind subjects to altitude, the system was also running normoxic airflow into the chamber during the NN sessions.

Each session consisted of 26 h of exposure in each condition (NN, NH, HH) in a randomized blinded order. After 24 h of exposure, subjects completed a self-paced 250 kJ time-trial, being free to increase/decrease the resistance to adjust the workload as they became familiar with it in the preliminary session (up/down electronic shifter at the handlebar). All trials were preceded by a rest period of 5 min, followed by 3 min warm-up period at 70 W. Setting (bike dimensions and braking resistance) was individualized but strictly identical between the three conditions for each subject. The participants were instructed to complete the 250 kJ as rapidly as possible. Work progressively completed from 0 to 250 kJ was the only information (visually) provided to subjects during the time-trial. With regard of the participants expertise in cycling and their VO_2_max, it was expected that the self-paced exercise would last 15–20 min in normoxia. In order to prevent excessive thermal stress, a fan providing a high wind speed was placed directly in front (∼80 cm) of the subjects during the trials.

Particular attention was given to the standardization and the control of the conditions. The participants were asked to maintain their usual training and physical activities during the whole experimental protocol to avoid fitness changes between sessions. Similar standardized meals were provided at the same time under each condition. The bedding was similar among conditions and sleep quality was not different between HH and NH (Saugy et al., 2016). The daily schedule and activities were exactly the same for all the conditions and the time trial was performed at the same time of the day in each condition.

### 2.3 Measurements

#### 2.3.1 Gas exchanges, heart rate, SpO_2_ and perceptual variables

Breath-by-breath pulmonary gas exchange (oxygen uptake, VO_2_; carbon dioxide production and end-tidal carbon dioxide partial pressure, P_ET_CO_2_), minute ventilation ( $\dot{V}$ E) and respiratory frequency (Rf) were measured at rest and throughout time-trial using a portable gas analyzer (MetaMax 3B, Cortex, Leipzig, Germany) with an oronasal mask (Vmask™, 7,500 series; Hans Rudolph Inc., Shawnee, United States). Heart rate (HR) and peripheral oxygen saturation (SpO_2_) were continuously recorded (Radical-7^®^, Masimo Corporation, Irvin, United States) and stored for offline analyzes with commercially available software (Labchart software, AD Instrument, Colorado Springs, United States). The VO_2max_ was not measured in hypoxic conditions but estimated by decrementing the NN VO_2max_ (*i.e.,* during the preliminary visit) by 22.7% following the 7.7% estimated linear VO_2max_ decrement per 1,000 m described by Wehrlin et al. (Wehrlin and Hallén, 2006).

Subjects were regularly asked to qualify their rate of perceived exertion (RPE, Borg Scale, 6–20) during the time-trial. Legs and breathing feelings were also assessed with visual analog scales (VAS, 0–10), ranging from “no difficulty” to “extremely difficult”.

#### 2.3.2 Power, workload and cadence

Power output, speed, cadence and total workload were continuously recorded during the time-trial by the cycle ergometer (Cycleops IC 400 Pro, Madison, United States).

#### 2.3.3 Electromyographic recordings

Quadriceps electromyography (EMG) was continuously recorded from the right *vastus lateralis* (VL) using bipolar silver chloride surface electrodes of 10-mm diameter (Kendall Meditrace 100). Electrodes were taped lengthwise on the skin over the muscle belly following SENIAM recommendations, with an interelectrode distance of 20 mm. Positions of the electrodes were marked on the skin to ensure precise replacement in other sessions. Reference electrode was attached on the patella. Low impedance (<10 kΩ) at the skin-electrode was obtained by shaving and abrading the skin with an abrasive sponge and cleaning with alcohol. EMG data were recorded at 2 kHz with Biopac system (MP150, Biopac System, Goleta, United States) and amplified with a bandwidth frequency ranging from 10 to 500 Hz. For data analysis, the integral of the EMG activity was calculated over 10-kJ time-periods throughout time-trial using the formula:

$$iEMG\;\left(\left|m\left(t\right)\right|\right)=\int_{0}^{1}\left|m\left(t\right)\right|dt$$

where *m* is the raw EMG signal.

#### 2.3.4 Cerebrovascular variables

Mean middle cerebral artery blood flow velocities (MCAv) were measured bilaterally using a 2-MHz pulsed Doppler ultrasound system (ST3, Spencer technology, Seattle, United States). The Doppler ultrasound probes were positioned over right and left temporal windows and held firmly in place with an adjustable headband (Marc 600 Headframe, Spencer technology). The signals were at depths ranging from 44 to 58 mm. Signal quality was optimized using an M-mode screen shot and insonation depth, probes and headband locations were marked to ensure within-subject repeatability. Bilateral MCAv were averaged to represent an index of global cerebral blood flow at rest and during exercise. Cerebral O_2_ delivery (cDO_2_) before and during exercise was calculated using the equation: cDO_2_ = mean MCAv x CaO_2_, where CaO_2_ refers to the oxygen content of the arterial blood estimated as follows: CaO_2_ = [hemoglobin concentration assessed in each condition after 20 h of exposure x 1.36 x current SpO_2_/100], oxygen dissolved in plasma being neglected. cDO_2_ was then expressed as a percentage of the resting normoxic (NN) pre-exercise value.

#### 2.3.5 Near-infrared Spectroscopy measurements

Cerebral oxygenation in the left prefrontal (PFC) and motor (MC) cortex was assessed by monitoring changes in oxy- and deoxy-hemoglobin (O_2_Hb and HHb, respectively) obtained with spatially resolved, continuous wave near-infrared spectroscopy (NIRS) (Oxymon MkIII, Artinis, Zetten, Netherlands). Theoretical and performance details of NIRS have been previously described (Perrey, 2008). PFC NIRS probes were centered between Fp1 and F3 locations according to the international 10–20 EEG system, with 3.5-cm interoptode distance. MC NIRS data were expressed as the average of a 4-channel square setting (3-cm interoptode distance) fixed with headbands between Cz and C3 locations. Muscle oxygenation was assessed from the right *vastus lateralis (at mid thigh)* using a 4-cm interoptode distance. For PFC and muscle, probe holders were secured to the skin using double-sided adhesive tape to minimize any change in its relative position and all optodes were covered with black sweatbands for them to be shield from ambient light. Total hemoglobin changes (THb = O_2_Hb + HHb) were calculated to reflect the changes in tissue blood volume within the illuminated areas and difference in hemoglobin (HbDiff = HbO_2_ - HHb) was calculated as a reliable estimator of change in tissue (de-) oxygenation status (Rooks et al., 2010). NIRS data were recorded at 10 Hz, filtered with a 2-s moving Gaussian window smoothing algorithm and expressed as relative changes (∆μmol) from the stable baseline preceding each time-trial.

### 2.4 Statistics

Data are reported as means and standard deviations with 95% confidence intervals. Data were tested for equality of variance (Fisher-Snedecor *F-test*) and for normality (Shapiro-Wilk test). To investigate the pacing strategies, we divided the time-trial in 25 slices of 10 kJ (increments of 4% of the total work completed). One-way ANOVA were used to determine if systemic (SpO_2_, VO_2_) and cerebrovascular (MCAv, cDO_2_) variables were different between conditions before the time-trial (*i.e.*, at baseline, BL). When a significant main effect was found, Bonferroni *post-hoc* tests were used to localize differences between conditions (NN, NH, HH). Two-way ANOVA (condition x time) with repeated measures were used for each parameter during the time-trial. When significant main or interaction effects were found, Bonferroni *post-hoc* tests were used to localize differences between conditions (NN, NH, HH) and/or time (each 10 kJ slice from 0 to 250 kJ). Null hypothesis was rejected at *p <* 0.05. All analyses were made using Sigmaplot 11.0 software (Systat Software, San Jose, United States).

## 3 Results

### 3.1 Time-trial performance and pacing

**FIGURE 1:**
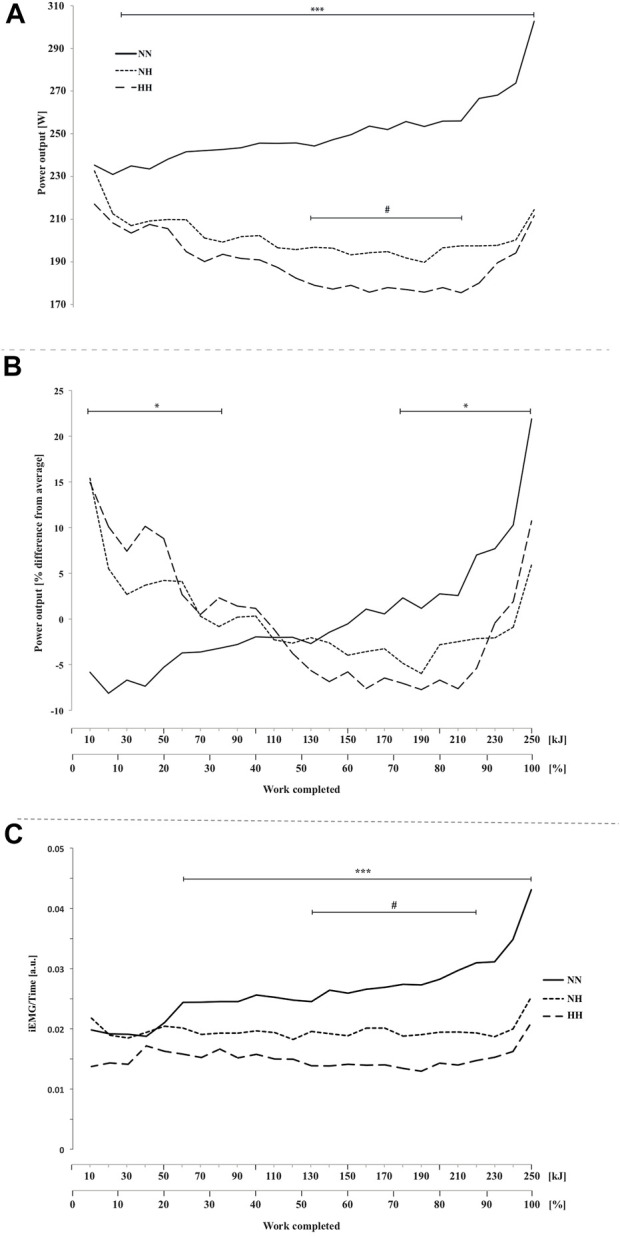
Power output **(A)**, normalized power output **(B)** and electromyographic activity (iEMG) measured for the *vastus lateralis* **(C)** during the self-paced 250-kJ time-trial in normobaric normoxia (NN), normobaric hypoxia (NH) and hypobaric hypoxia (HH). *p < 0.05, ***p < 0.001 for difference between NN and both hypoxic conditions; ^#^ *p* < 0.05 for difference between NH and HH.

Time-trial performance data are summarized in Table 1. Compared to NN (*i.e.*, 1,041 ± 151 [955.9–1,126.9] s), the mean time required to complete 250 kJ was 24.1 ± 9.6 [18.7–29.5] % and 33.2 ± 12.4 [26.2–40.2] % higher for NH and HH, respectively (both *p* < 0.001, cf. Supplementary Material). The mean time was 7.5 ± 7.5 [3.2–11.7] % higher in HH than in NH (*p* < 0.01). Compared to NN the whole time-trial pace was reduced for both hypoxic conditions (Figure 1A, *p* < 0.001). The HH power output was significantly reduced compared to NH from 140 to 220 kJ (*p* < 0.05). When expressed as a function of the average power sustained in each respective condition (Figure 1B), an interaction effect was observed (*p* < 0.05) showing inverse trends between NN and both NH and HH, despite a comparable range of variation (∼30%, ∼21 and ∼23%, for NN, NH and HH, respectively). In HH, normalized power output followed a similar pattern to NH. Between 85% and the end of the time-trial (so called “final burst” or “end-spurt”), the relative increase in power output was 9, 18 and 21% in NH, HH and NN, respectively.

**TABLE 1:** Baseline physiological measurements before exercise and performance results during the self-paced 250-kj time-trial in normobaric normoxia (NN), normobaric hypoxia (NH) and hypobaric hypoxia (HH). ***p < 0.001 for difference between NN and both hypoxic conditions; ##*p* < 0.01 for difference between NH and HH.

	Normobaric normoxia	#auto; normobaric Hypoxia	#auto; Hypobaric Hypoxia
#auto; Baseline Measurements			
#auto; *Sp O 2 (%)*	98 ± 1 ***	92 ± 2	90 ± 2 ##
#auto; *HR (bpm)*	69 ± 11 ***	77 ± 12	81 ± 8
#auto; Time-trial Performance data			
#auto; *Mean duration (s)*	#auto; 1,041 ± 151 ***	#auto; 1,286 ± 167	#auto; 1,379 ± 187 ##
#auto; *Mean Power (W)*	#auto; 245 ± 39 ***	#auto; 197 ± 26	#auto; 184 ± 25
#auto; *Mean cadency (rpm)*	#auto; 95 ± 7	#auto; 93 ± 9	#auto; 88 ± 6 #

### 3.2 Pulse oxygen saturation

**FIGURE 2:**
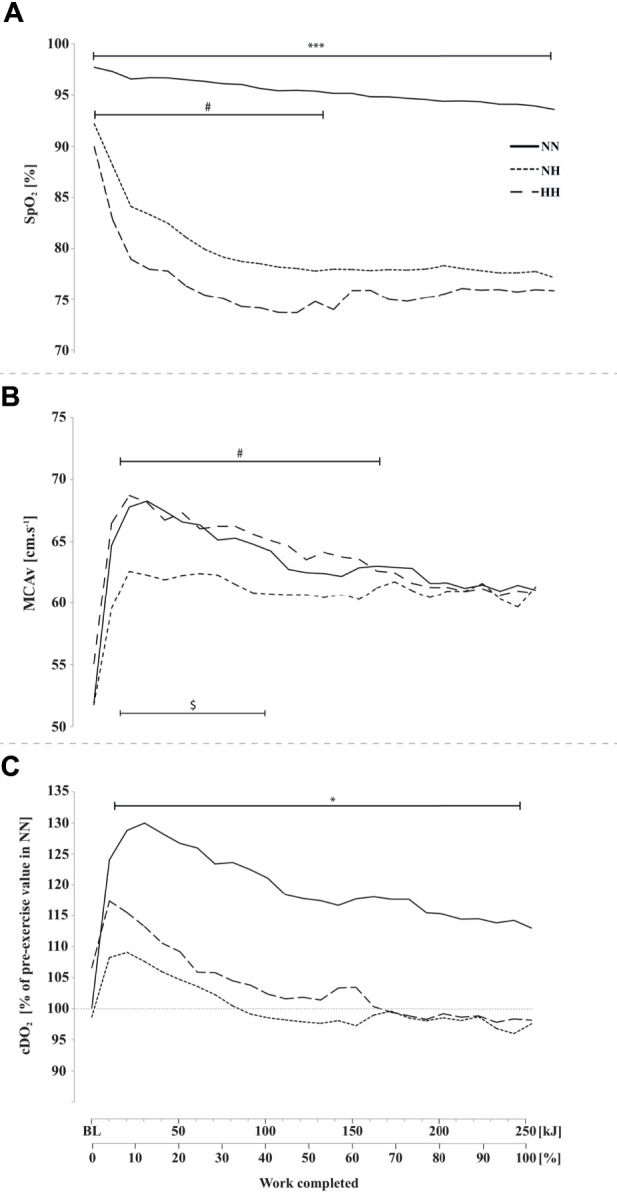
Peripheral oxygen saturation (SpO_2_, panel **(A)**, middle cerebral artery blood flow mean velocity (MCAv, panel **(B)** and cerebral oxygen delivery cDO_2_, panel **(C)** during the self-paced 250-kJ time-trial in normobaric normoxia (NN), normobaric hypoxia (NH) and hypobaric hypoxia (HH). *p < 0.05, ***p < 0.001 for difference between NN and both hypoxic conditions; ^$^ *p* < 0.05 for difference between NN and NH; ^#^ *p* < 0.05 for difference between NH and HH.

As presented in Figure 2A, SpO_2_ was significantly higher at baseline (97.7 ± 1.2 [97.1–98.4] %; *p* < 0.001) and during cycling in NN compared to both hypoxic conditions (0–250 kJ; *p* < 0.001) and SpO_2_ was significantly lower in HH compared to NH at baseline (92.2 ± 2.1 [91.1–93.3] vs 89.9 ± 1.9 [88.9–91.1] %; *p* < 0.01) and during the first half of the time-trial (0–140 kJ; *p* < 0.05).

### 3.3 Cerebrovascular variables

MCAv at baseline was not different between conditions. MCAv was increased in the first quarter of the time-trial in both NN, NH and HH (+31%, +15% and +25%, respectively), the increase being lower in NH compared to HH (from 10 to 150 kJ, *p* < 0.05) and NN (from 10 to 100 kJ, *p* < 0.05) while no difference was found along time-trial between HH and NN (Figure 2B), where MCAv decreased from 40 kJ onward. Baseline cDO_2_ values were not different between conditions (Figure 2C). In contrast, during time-trial cDO_2_ was lower in both NH and HH (on average, by 17%) compared to NN (*p* < 0.05), with no significant difference between NH and HH. In the latter conditions cDO_2_ decreased to near baseline values at ∼100 kJ while it remained elevated in NN (*p* < 0.05).

### 3.4 Cardio-respiratory parameters

**FIGURE 3:**
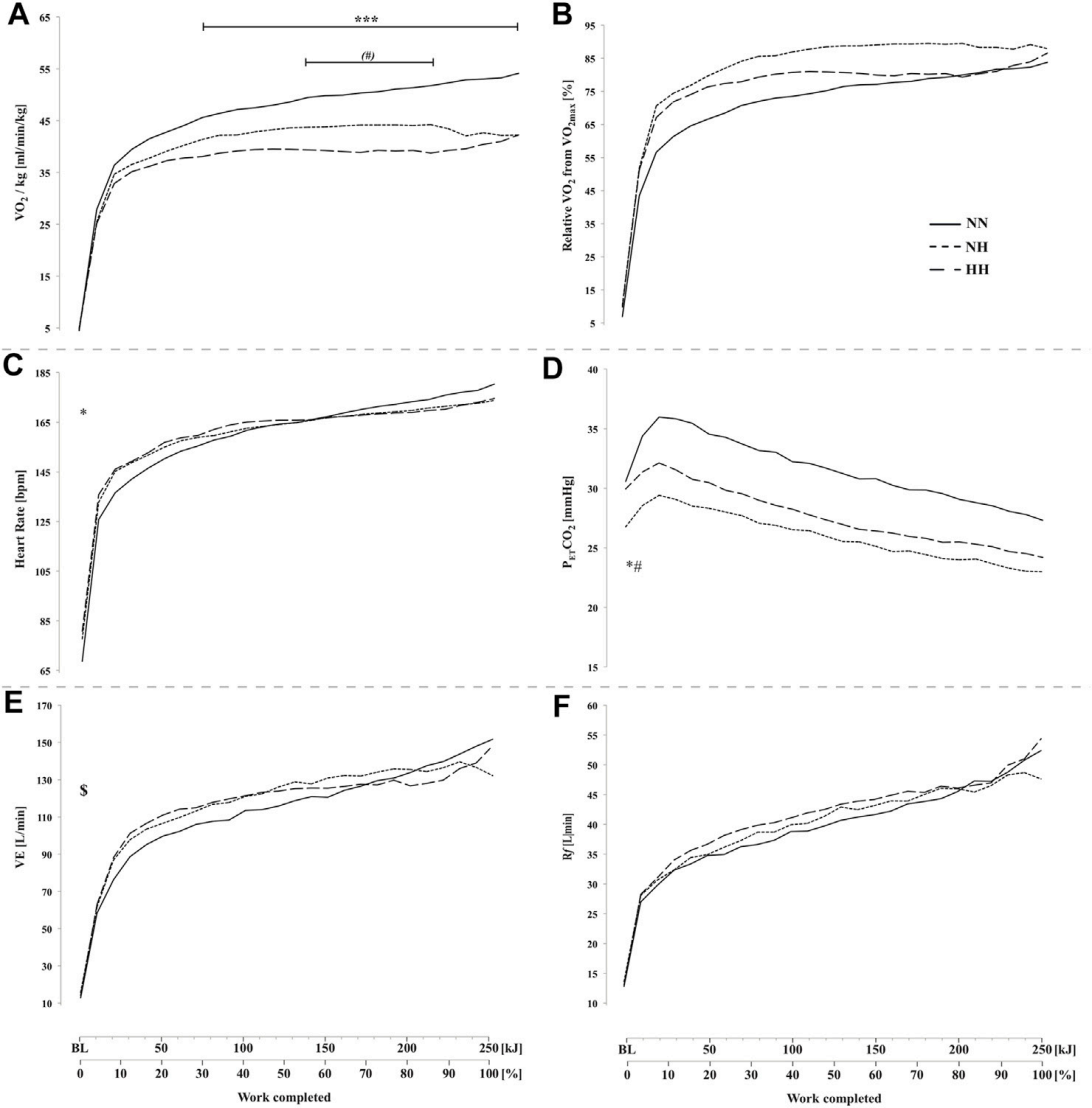
Absolute oxygen uptake VO_2_, panel **(A)**, oxygen uptake relative to VO_2max_ panel **(B)**, heart rate (panel **(C)**, end-tidal CO_2_ pressure (P_ET_CO_2_, panel **(D)**, minute ventilation ( $\dot{V}$ E, panel **(E)** and respiratory frequency (R*f*, panel **(F)** during the self-paced 250-kj time-trial in normobaric normoxia (NN), normobaric hypoxia (NH) and hypobaric hypoxia (HH). *p < 0.05; ***p < 0.001 for difference between NN and both hypoxic conditions; ^$^ *p* < 0.05 for difference between NN and NH; ^(#)^ *p* = 0.08; ^#^ *p* < 0.05 for difference between NH and HH.

VO_2_ was higher for NN compared to both hypoxic conditions (from 70 to 250 kJ; *p* < 0.001) and trend to be lower for HH compared to NH before the end-spurt (from 140 to 210 kJ; *p* = 0.08) (Figure 3A). No difference was observed between conditions in VO_2_ relative to VO_2max_ (Figure 3B). Heart rate was higher at baseline for both hypoxic conditions (78 ± 12 [71–84] and 81 ± 8 [76–85] bpm for NH and HH, respectively) compared to NN (69 ± 11 [62–75] bpm) but no difference was observed during the time-trial (Figure 3C). P_ET_CO_2_ at baseline was lower for NH (26.8 ± 2.9 [25.1–28.4] mmHg) than NN (30.6 ± 3.3 [28.7–32.5] mmHg; *p* < 0.05) and HH (29.9 ± 2.5 [28.5–31.4] mmHg; *p* < 0.05), but no statistical difference was reached during the time-trial (Figure 3D). $\dot{V}$ E was higher in NH than NN at baseline (15.8 ± 5.2 [12.8–18.8] and 12.8 ± 4.2 [10.4–15.1] L.min^−1^, respectively, *p* < 0.05) but no difference was found between NH and HH at rest or between conditions during exercise (Figure 3E). No difference was found neither at baseline, nor during exercise for Rf (Figure 3F).

### 3.5 Cerebral oxygenation

**FIGURE 4:**
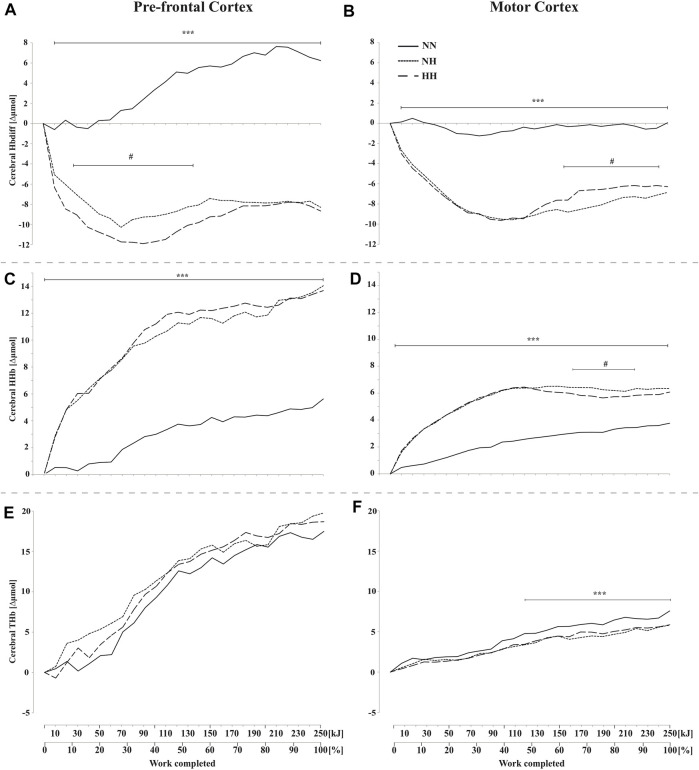
Mean changes in cerebral hemoglobin difference (HbDiff = O_2_Hb-HHb), deoxy-(HHb), and total-hemoglobin (THb), during the self-paced 250-kJ time-trial in normobaric normoxia (NN), normobaric hypoxia (NH) and hypobaric hypoxia (HH). Data are shown for the prefrontal cortex (PFC, panels **(A,C,E)**) and for the motor cortex (MC, panels **(B,D,F)**). ***p < 0.001 for difference between NN and both hypoxic conditions; ^#^ *p* < 0.05 for difference between NH and HH.

These results are presented in Figures 4A–F. PFC and MC HbDiff were higher for NN than for the two hypoxic sessions throughout the time-trial (*p* < 0.001, Figures 4A,B). Lower values were found in HH compared to NH in PFC HbDiff at the beginning of the time-trial (20–120 kJ, *p* < 0.05, Figure 4A), while higher values were observed in HH compared to NH in MC HbDiff in the second part of the time-trial (160–240 kJ, *p* < 0.05, Figure 4B). Exercise induced an increase in cerebral HHb that was lower for NN than the two hypoxic conditions in both PFC and MC (*p* < 0.001, Figures 4C,D). This increase was particularly limited during the first half of the time-trial in NN. During the second half of the time-trial, both MC HbDiff and HHb tended to “plateau”. MC HHb was also slightly lower for HH compared to NH in this last part of exercise (*p* < 0.05). Increase in cerebral THb along exercise was similar in the three conditions in PFC (Figure 4E) but was higher for NN in MC during the second half of the time-trial (from 100 kJ onward, *p* < 0.001, Figure 4F) compared to the increase observed in both hypoxic conditions.

### 3.6 Muscle oxygenation

**FIGURE 5:**
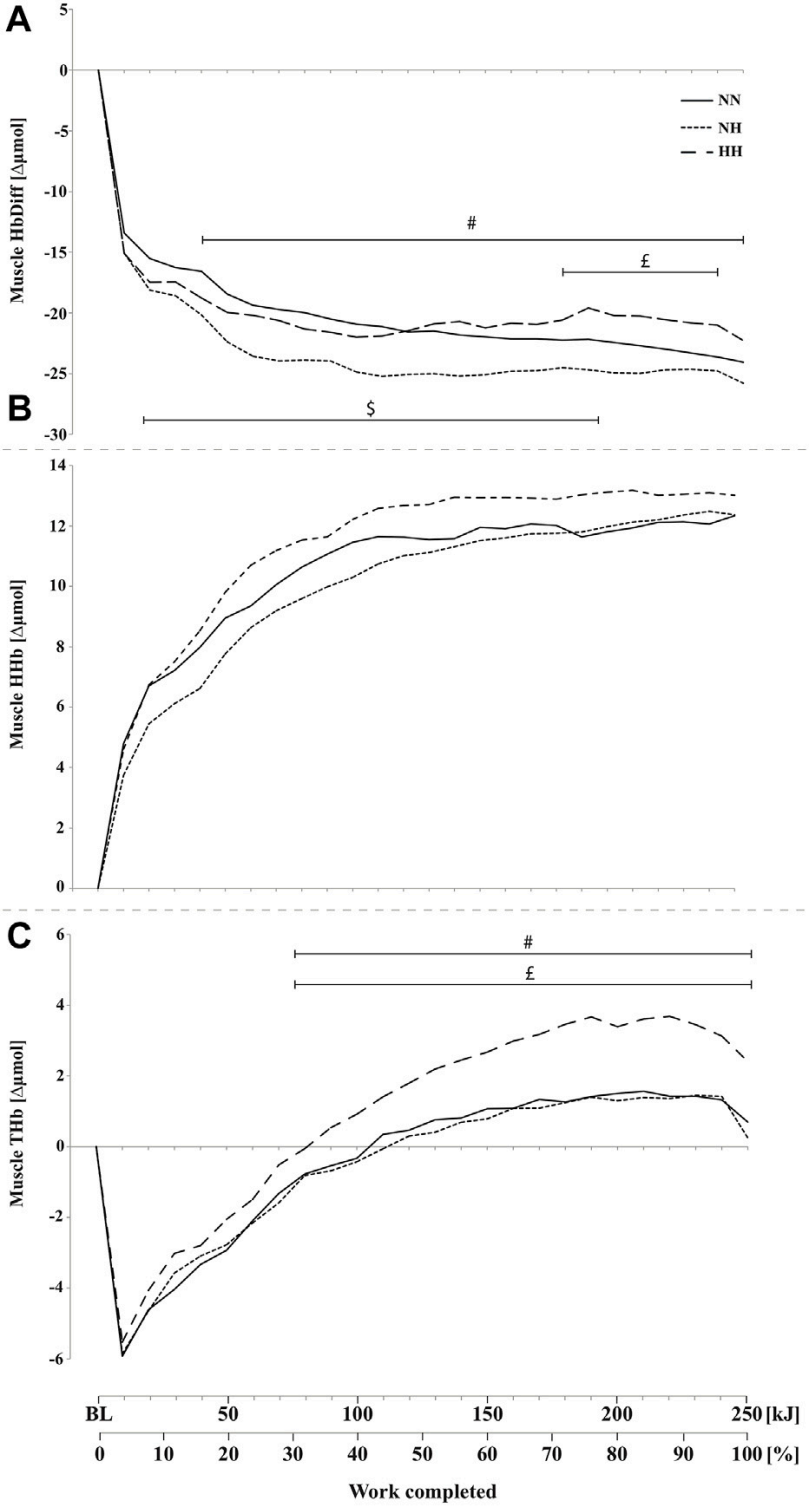
Mean changes in muscle hemoglobin difference (HbDiff = O_2_Hb-HHb, panel **(A)**, deoxy-(HHb, panel **(B)**, and total-hemoglobin (THb, panel **(C)**, during the self-paced 250-kJ time-trial in normobaric normoxia (NN), normobaric hypoxia (NH) and hypobaric hypoxia (HH) for the *vastus lateralis*. ^$^ *p* < 0.05 for difference between NN and NH; ^£^ *p* < 0.05 for difference between NN and HH; ^#^ *p* < 0.05 for difference between NH and HH.

Muscle HbDiff was lower for NH compared to NN from 20 to 210 kJ (*p* < 0.05) and lower for NN compared to HH from 190 to 240 kJ (*p* < 0.05) (Figure 5A). HbDiff was also lower in NH compared to HH from 50 kJ onward (*p* < 0.05). No difference has been found in muscle HHb increase during time-trials between conditions (Figure 5B). Muscle THb was higher in HH compared to both NN and NH conditions from 90 kJ onward (*p* < 0.05) (Figure 5C), with no difference between NN and NH.

### 3.7 Electromyographic activity

EMG activity was higher for NN compared to both hypoxic conditions from 60 kJ onward (Figure 1C, *p* < 0.001) and it was lower for HH compared to NH from 130 to 210 kJ (*p* < 0.05).

### 3.8 Subjective feelings

**FIGURE 6:**
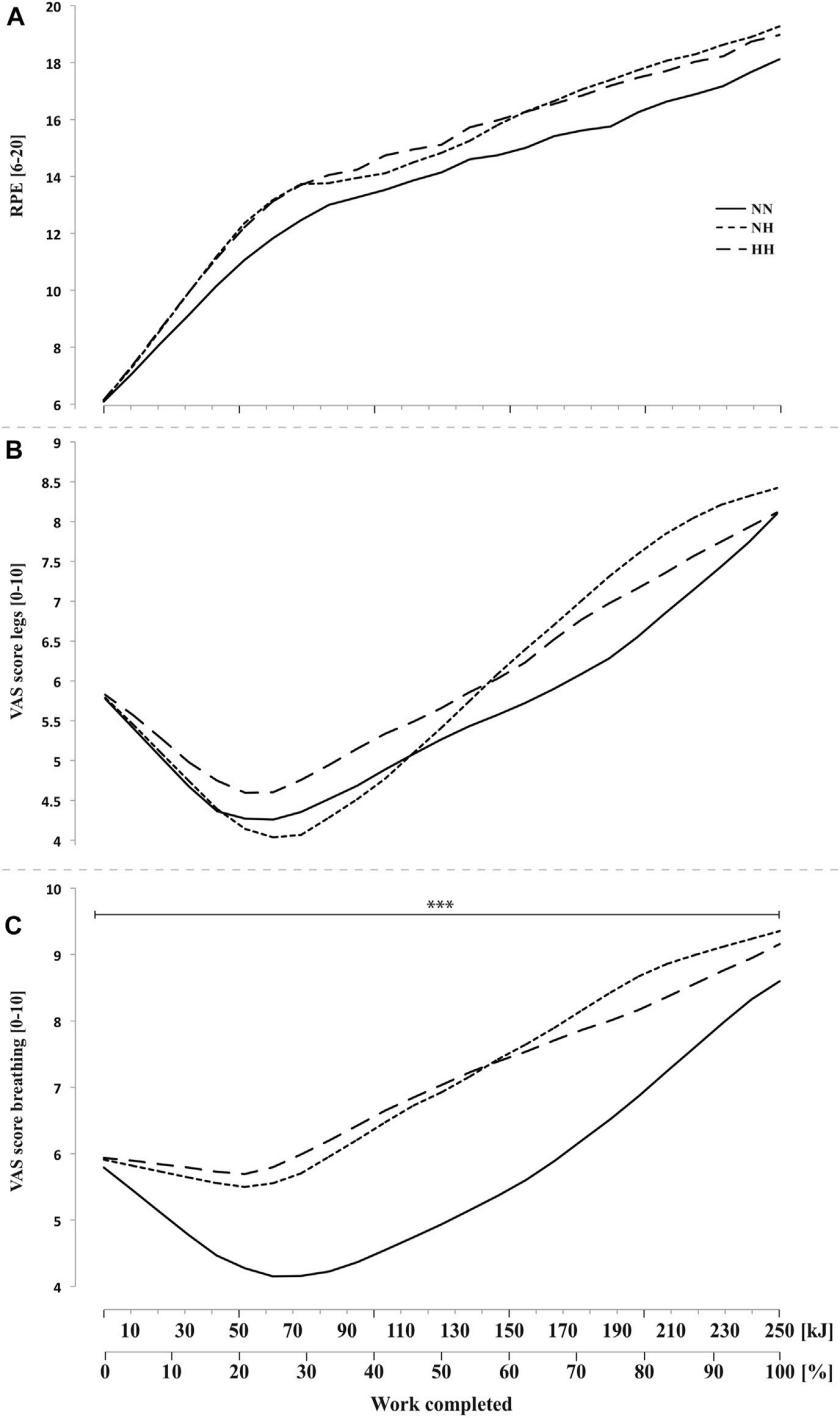
Rate of perceived exertion (RPE, panel **(A)** and visual analog scale (VAS) scores for legs feelings (panel **(B)** and breathing discomfort (panel **(C)** during the self-paced 250-kJ time-trial in normobaric normoxia (NN), normobaric hypoxia (NH) and hypobaric hypoxia (HH). ***p < 0.001 for difference between NN and both hypoxic conditions.

Perceived exertion (Figure 6A) and legs discomfort (Figure 6B) were not different between conditions along cycling. However, breathing discomfort (Figure 6C) was higher for both hypoxic conditions from the beginning to the end of the time-trial (*p* < 0.001, with no difference between NH and HH. In addition, a biphasic evolution from 0 to 70 kJ (i.e. improvement or plateau) and then from 70 to 250 kJ (i.e. worsening) was observed with a time effect on both legs and breathing discomfort (both *p* < 0.001, Figures 6B,C).

## 4 Discussion

This study was designed to investigate time-trial performance in conjunction with systemic, muscle and cerebrovascular variables after a 24-h exposure to hypoxia. The major findings were that, for the same pressure in oxygen, there were subtle differences in cerebrovascular regulation during an endurance exercise performed in normobaric *versus* hypobaric hypoxia likely resulting in differences in pacing and total performance. Moreover, there was an inverse and more variable pacing pattern in hypoxia compared to normoxia, in conjunction with depressed cerebrovascular function for a same relative intensity of exercise (*i.e.*, %VO_2max_, HR, $\dot{V}$ E, VO_2_, RPE).

### Pacing in hypoxia versus normoxia

Hypoxia-induced impairments of aerobic performance have been extensively studied by the past but scarce studies have described how pacing strategy (*i.e.*, self-selected distribution of power output or energetic reserves) may help to understand why we fatigue when oxygen availability becomes challenging.

Due to the experimental design, participants started exercise at the same workload (∼226 W) in normoxia and hypoxia but acutely reduced power output until end-spurt in hypoxia whereas a slight positive trend was seen in normoxia over the same period. Variation in power outputs (as a function of mean power during the trial) from the start to 85% of time-trial was twice higher in hypoxia compared to normoxia (∼22 and ∼11%, respectively).

Previous reports examined exclusively time-trials performed after very short exposure to hypoxia, ranging from only 3–40 min (Clark et al., 2007; Fan and Kayser, 2013; Périard and Racinais, 2016) up to 2 h (Beidleman et al., 2014). Sudden and acute hypoxic exposure provides an interesting model that stresses oxygen transport systems but in real-world training/competing (particularly at terrestrial altitude), exposure time preceding exercise is usually longer and is prone to deeply influence how pace would be regulated (Tucker, 2009). We previously showed that the first hours at altitude progressively affect motor cortical excitability (Rupp et al., 2013a) or muscle and cerebral oxygenation (Rupp and Leti, 2013) for instance. In the present experiment, the prooxidant/antioxidant balance became impaired from 10 h in hypoxia (Ribon et al., 2016), hematologic parameters were affected after 20 h in hypoxia (Saugy et al., 2016) and sleep quality was significantly disturbed in hypoxia the night before time-trial (Heinzer et al., 2016). This might, at least partly explain why available evidence suggests that the acute reduction in both maximal aerobic power and endurance performance is maximal within 16–24 h of exposure (Schuler et al., 2007), and resorbs progressively thereafter.

***Initial phase.*** The intensity set at the start of the time-trial in hypoxia led to a rapid decrease in power output over that period (from start to kJ 70), with a 15% decrease in normalized power output compared to a +3% variation only in normoxia (Figure 1A). Interestingly, similar rate of increase in RPE were observed in all conditions in the present study in the first 30% of completed work (0–70 kJ). These RPE were however generated from a very different balance between central drive and afferent feedbacks (physiological adaptations) in normoxia and hypoxia. A wide range of variables can influence pacing but recently, tactical adaptations in the chosen pacing strategies have been incredibly manipulated with the use of central nervous system drugs and selective block of the central projection of ascending sensory pathways (*e.g.*, fentanyl, opioid analgesic) (Amann et al., 2009), so that the understanding of the role of neurophysiological processes has grown.

Cortical representation of muscle involved in cycling is served dominantly by MCA (Jorgensen, Perko and Secher, 1992). Transcranial Doppler can provide quantitative information on cerebral hemodynamic changes at the macrovascular level (*i.e.*, cerebral arteries) but is unable to assess directly the qualitative repercussions of such changes for the tissue at the microvascular level. How much reductions in SpO_2_ and cDO_2_ translate into changes in cerebral tissue oxygenation when self-paced exercise is performed in hypoxia remains largely unknown. Hence, NIRS is increasingly used to measure the (mis)balance between oxygen supply and utilization directly in tissue micro-vessels (venules, arterioles and capillaries), with a predominant venous contribution (70–80%) (Hamaoka et al., 2007).

We found that the cerebrovascular responses in NN over the first part of exercise appeared appropriate (*e.g.*, cDO_2_ maintained at ∼125% from baseline value, preserved/increased brain oxygenation), while it is likely that the brain function was threatened in hypoxia over that period from a rapid decrease in SpO_2_, cDO_2_ (Figures 2A,C) and cerebral oxygenation towards low levels (Figures 3A–D).

***Main phase.*** From 30 to 85% of the time-trial mean power output difference between NN and both hypoxic conditions continued to grow (Figure 1A), still without any difference in RPE (Figure 6) and relative exercise intensity as expressed from %VO_2max_ (Figure 3B) HR (Figure 3C) or ventilation parameters (Figures 3E,F). The lower work-rate in hypoxia was achieved by the alteration in the degree of skeletal muscle recruitment (*i.e.*, lower iEMG) (Figure 1C) in accordance with Peltonen et al. (Peltonen et al., 1997). This is corroborated for the first time by a concomitant significant cerebral hypoperfusion at the micro-vascular level (from THb with NIRS, Figure 4F) over the motor cortex, which directly drives the muscles.

Synthesizing information from a wide range of brain systems and exerting control over cognitive and executive behavior (*e.g.* sensory information integration, decision-making, movement planning, pacing strategies and motivation), PFC associative areas play a central role in the orchestration of thoughts and actions in accordance with internal goals (Ramnani and Owen, 2004) (of particular interest in our study), but the central motor drive is ultimately conducted from the premotor and primary motor areas, which have never been investigated during self-paced exercise before. From the study of Subudhi et al. (Subudhi et al., 2009) it has been often argued that there is a good correlation between prefrontal, premotor and motor cortices oxygenation measurements during exercise. However, this has been shown during a short, maximal incremental exercise, where pacing strategy was minimal as power output was compelled throughout the test. We recently demonstrated that PFC and MC oxygenation profiles can differ during submaximal fatiguing exercise (Rupp et al., 2013b) and to our knowledge the present study is the first to present simultaneous macro-circulation in MCA and both PFC and MC micro-hemodynamics and oxygenation during a self-paced exercise.

Here, PFC and MC oxygenation were both markedly depressed in hypoxia from the start of the time-trial (Figures 4A–D), confirming what has been reported before almost exclusively during progressive maximal exercises in hypoxia and in the PFC (Subudhi et al., 2009; Vogiatzis et al., 2011; Marillier et al., 2021). Presumably, the traditionally-observed exercise-induced increase in cerebral cortex oxygenation reflects progressive increase in oxygen metabolic demand with increased neuronal networks activation. Despite lower central drive and muscle activity during time-trial in hypoxia, it is likely that the challenging environmental conditions (cf., low F_i_O_2_) blunted the ability of the neurovascular coupling to increase or even maintain cerebral oxygenation to habitual levels aiming at preserving a positive balance between oxygen supply and consumption. An hypothesis might be that power output is rapidly then steadily decreased in hypoxia to prevent the body to be exposed to unacceptable levels of SpO_2_, cDO_2_ and cerebral oxygenation or, at least, to values that would be considered as incompatible with the remaining expected time before exercise completion. The diminished power output certainly allowed cDO_2_ and cerebral hemodynamics not to decrease further (e.g. under baseline values for cDO_2_). Of interest is that both PFC and MC HbDiff plateaued in hypoxia during the second half of the time-trial while the rate of HHb increase was lower. On the other hand it is important to stress that PFC and MC oxygenation would be only one of many afferent signals influencing complex regulation of motor drive during self-paced exercise (St Clair Gibson and Noakes, 2004).

In the present study, CBF declined in both NN and HH despite increased *versus* decreased power output, respectively. This adaptation mirrors the P_ET_CO_2_ decrease over the same period (Figure 3D) and might thus be triggered by hyperventilation-induced hypocapnia (cf. Figures 3E,F). This assumption is corroborated by previous results who observed a similar decrease in MCAv during a 750-kJ time-trial in normoxia (Périard and Racinais, 2016). Conversely, other results demonstrated a maintain MCAv (in normoxia) or an increased MCAv (and almost maintained cDO_2_) in hypoxia during a 15-km time-trial (Fan and Kayser, 2013). To explain these results, authors underlined a higher RPE during the time-trial in hypoxia (5,000 m) and suggested a greater sensorimotor activation compared to normoxia.

***End-spurt.*** In accordance with well-known field observations and as previously described in the literature (Roelands et al., 2009) our results indicated a characteristic end-spurt phenomenon in the last 10–15% of the time-trial. This end-spurt was seen whatever the condition (Figure 1B). Our results confirm that the subjects have the drive and/or motivation to augment power output when approaching the end-point in normoxia but also in hypoxia (what is not the case anymore after administration of a serotonin reuptake inhibitor in normoxia) (Roelands et al., 2009).

### Normobaric versus hypobaric hypoxia

When comparing HH and NH, it should be noted that power output at the immediate onset of the time-trial (0–20 kJ, 8%) was similar and also similar to NN, suggesting that the initial selection of work rate was more likely based on previous experience and expectations of exercise duration, rather than on an instantaneous (baseline) afferent input from hypoxemic or disturbed organ/tissue homeostasis (*e.g.*, modified baseline HR, SpO_2_, P_ET_CO_2_). Indeed, even the knowledge of the condition (no possibility to blind HH) had no influence on the initial power output, and we recall participants had no feedback on their power output throughout the time-trials. However, the tendency towards lower power output in HH in the first part of the time-trial appeared significant in the second part (from ∼30% of total work onward). Trends and normalized variation in power output (Figure 1A) were similar in HH and NH throughout the time-trial, but for the first time we were able to identify subtle differences in underlying physiological adaptations (i.e., cerebrovascular responses) with a potential explanation of the lower performance in HH.

Part of the explanation may arise from distinct baseline status in HH and NH after the 24-h exposure; time-window of particular interest with regards to the early signs of AMS for instance (Subudhi, Panerai and Roach, 2010), which are known to be correlated to the degree of hypoxemia (Fulco et al., 2011). Accordingly, baseline SpO_2_ before the time-trial was significantly lower in HH despite similar P_i_O_2_ (Figure 2A). This might have impacted subjective feelings (e.g. RPE, leg and ventilation discomforts) during exercise so that power output had to be further reduced in HH compared to NH to match an equivalent relative exercise intensity (%VO_2max_, HR, …). Besides, underlying mechanisms associated with the same relative exercise intensity showed differences, likely explaining lower power output in HH in the second half (140–220 kJ, corresponding to 55–85%) of the time-trial duration.

Mass oxygen delivery to the brain is dependent on cerebral blood flow (cDO_2_ assessed from MCAv in the present study) and on arterial content in oxygen, which is a function of hemoglobin concentration and arterial saturation in oxygen. It seems consistent from the literature to report that the rate of cDO_2_ is an important information integrated by the central nervous system to regulate central drive. We demonstrated that cDO_2_ was extremely reduced in both HH and NH compared to NN, already in the first part of the time-trial. It is particularly interesting to see how the progressive impairments in power output mirrored the decrease and plateau in cDO_2_, conceivably preventing any further decrease under dramatically low values in hypoxia. This was observed in a very similar extent in both HH and NH but we show here for the first time that the comparable cDO_2_ levels achieved, resulted from distinct mechanisms in NH and HH (cf. SpO_2_-MCAv profiles) and were seen in conjunction with significantly different cerebral oxygenation states in both conditions.

First SpO_2_ was significantly lower at rest and during the first half of the time-trial in HH compared to NH, what may explain *1*) the higher hemoconcentration measured before exercise in HH (+6%, (Saugy et al., 2016)) as a compensatory mechanism and, *2*) the higher MCAv values in HH (+7.5% over 10–150 kJ) throughout exercise likely due to a higher hypoxia-induced cerebral vasodilation (Casey and Joyner, 2012). At the same time, exercise-induced decrease in P_ET_CO_2_ was approximately the same in NN, NH and HH (-8 mmHg on average over 20–250 kJ with similar $\dot{V}$ E and Rf kinetics, Figure 3D–F), but this decrease started from a significant hypocapnic state only in NH (*e.g.* significantly lower value of P_ET_CO_2_ at baseline). Accordingly, MCAv appeared to be much more affected by hypocapnia-induced vasoconstriction in NH compared to HH (Figure 2B). Altogether, cDO_2_ was maintained at comparable levels in NH and HH, but likely from significantly lower values of CaO_2_ (despite a slight hemoconcentration) and significantly higher values of MCAv in HH.

However, impaired cDO_2_ is only part of the equation (*i.e.*, reduction in the ability of CNS to voluntarily activate skeletal muscle, (Goodall, Twomey and Amann, 2014)) and explains mainly the decreased performance in both NH and HH compared to NN. Cerebral oxygenation kinetics described in the present study also help to understand how and why pacing might be more down-regulated in HH compared to NH. From our data, one may speculate that a comparable central drive was produced in HH and NH in the initial part of the time-trial (*e.g.*, same MC deoxygenation, iEMG, power output, muscle deoxygenation, absolute VO_2_). However, PFC deoxygenation was significantly higher in HH (Figure 4A) during that period. As cDO_2_ was the same in HH and NH along the time-trial, the lower PFC oxygenation may have resulted from higher extraction rate of oxygen (*i.e.*, higher neuronal activity) in this part of the brain. Whatever the explanation (*e.g.*, greater integrative process, higher planning activity), the fact is that lowering power output in HH allowed PFC HbDiff to progressively “restore” to NH levels. Finally, these observations explain similar PFC activity in the second part of the time-trial for lower power output in HH than in NH. Consistently (from 55 to 85% of the total duration, prior end-spurt), the lower power output in HH *vs*. NH was seen in conjunction with a lower degree of muscle recruitment (*i.e.*, lower MC HHb, lower muscle deoxygenation and blood perfusion, lower iEMG).

### Methodological considerations

Some limitations inherent to NIRS measurement should be noted. Part of the detected NIR light may be affected by the changes in optical properties of superficial tissue layers between the optode and the investigated tissue (*e.g.* scalp and skull for the brain; skin and fat for the muscle) (Takahashi et al., 2011). We sought to minimize the effects of near-surface blood flow in the observed chromophore concentration changes by controlling room air temperature, by giving attention to ensure NIRS setup to be non-compressive and by using enlarged inter-optode distances to reach the maximal light path providing a sufficient signal-to-noise ratio of the optical density measurements (Rolfe, 2000). Moreover, CBF may be heterogeneously distributed at exercise and under hypoxia (Pagani et al., 2011), we investigated MCA macrocirculation plus multiple sites of interest by NIRS and we acknowledge that observed tissue oxygenation cannot be generalized to whole brain (or to other muscles from NIRS on *vastus lateralis*). Finally, we contend that MCAv is a reliable index of changes in global cerebral blood flow during exercise in normoxia and hypoxia, as the cross-sectional area of the MCA has been shown unchanged within a wide range of changes in P_ET_CO_2_ (Valdueza et al., 1997) and in comparable hypoxic situations (<5,000 m) (Poulin and Robbins, 1996).

## Conclusion

This study showed that pacing strategy during a cycling time-trial is impaired after 24 h in hypoxia (different trend and higher variability) compared to normoxia and this is likely the result from a compromised ability of the central nervous system to voluntarily activate skeletal muscles, owing to inadequate oxygen delivery to the brain. With simultaneous multi-systemic parameters, cerebrovascular function, muscle activity and subjective feelings, light is shed on tissue-specific adaptations and new insights are provided into the mechanisms underlying the higher pacing down-regulation in hypobaric *versus* normobaric hypoxia. Despite equivalent PiO_2_, HH is a more stressful stimulus than NH (*e.g.*, lower SpO_2_ and higher PFC deoxygenation for a given power output during the first half) and suggests a higher “adaptive cost” (same RPE, leg and ventilation discomforts for a lower power output during the second half). As a consequence, aiming to maintain an equivalent oxygen delivery to the brain, the system likely adopts a more “protective” strategy, leading to a further impaired performance in HH. In addition, same relative exercise intensity and physiological disturbances were achieved in HH from the production of lower absolute power when compared to NH, emphasizing once more time that exercise at terrestrial and simulated altitude cannot be carelessly interchanged.

## Data Availability

The raw data supporting the conclusions of this article will be made available by the authors, without undue reservation.
