# Supplementary figures and images for: Brain-muscle interplay during endurance self-paced exercise in normobaric and hypobaric hypoxia
Source: Front Physiol. 2022 Aug 25;13:893872. doi: 10.3389/fphys.2022.893872 (PMC9453479; doi:10.3389/fphys.2022.893872)

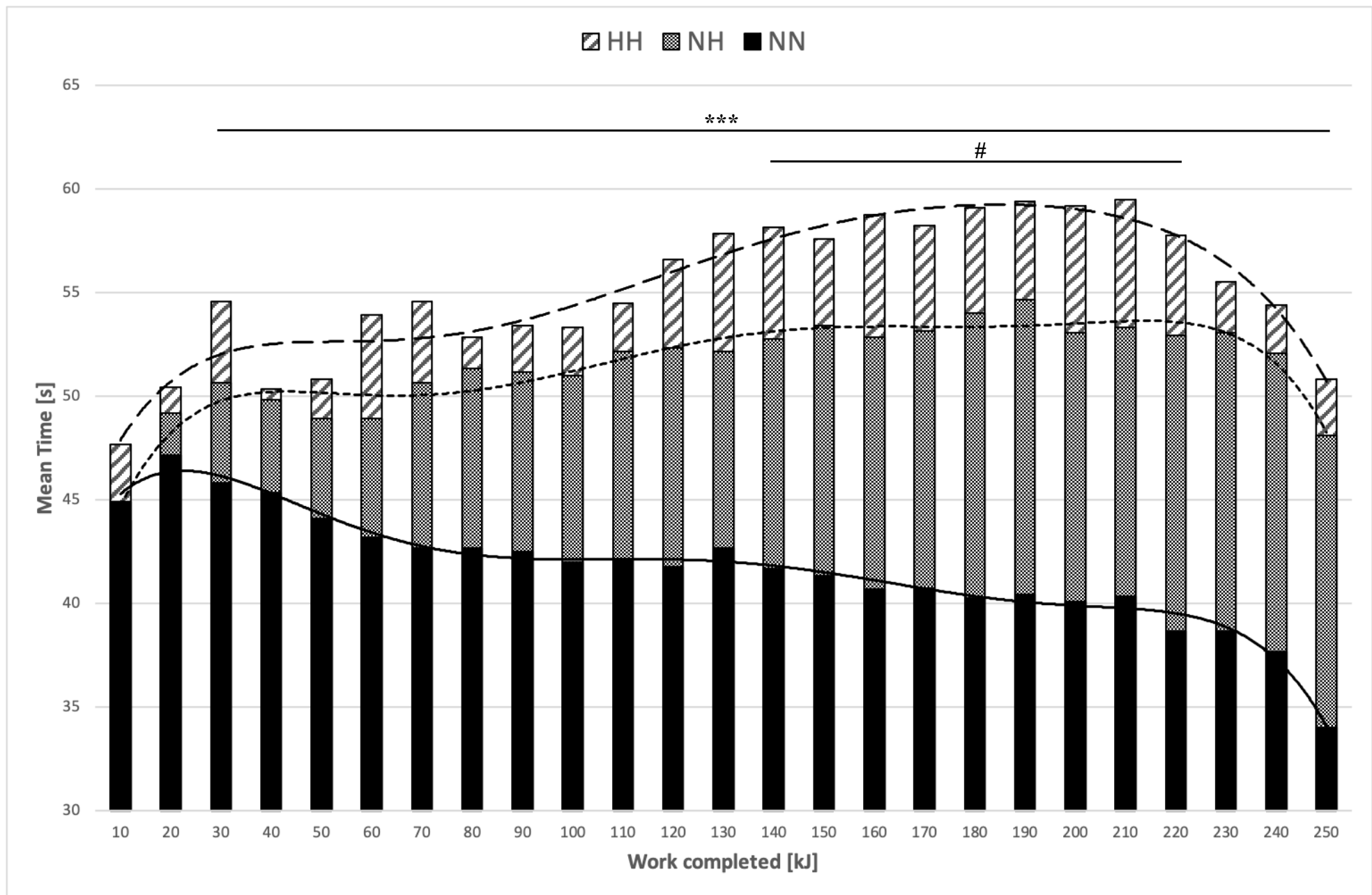

Supplement: Supplementary file 1 [file DataSheet1.PDF]
